# Effect of transcutaneous electrical nerve stimulation of acupoints on respiratory outcomes of COVID‐19 patients with moderate pulmonary involvement: A parallel randomized clinical trial

**DOI:** 10.1002/hsr2.1427

**Published:** 2023-07-22

**Authors:** Amin Shahdad, Nasrin Fadaee Aghdam, Shahrbanoo Goli, Ehsan Binesh, Javad Nourian, Mahboobeh Khajeh

**Affiliations:** ^1^ Student Research Committee, Nursing and Midwifery School Shahroud University of Medical Sciences Shahroud Iran; ^2^ Department of Nursing, School of Nursing and Midwifery Shahroud University of Medical Sciences Shahroud Iran; ^3^ Department of Epidemiology, School of Public Health Shahroud University of Medical Sciences Shahroud Iran; ^4^ Clinical Research Development Unit, Imam Hossein Hospital Shahroud University of Medical Sciences Shahroud Iran

**Keywords:** COVID‐19, mechanical ventilation, oxygen saturation, survival, transcutaneous electric nerve stimulation

## Abstract

**Background and aims:**

Experiencing respiratory symptoms, especially dyspnea and decreased oxygen saturation (SpO_2_) level in patients with coronavirus disease 2019 (COVID‐19) is associated with increased mortality. The present study was conducted to investigate the effect of transcutaneous electrical nerve stimulation of acupoints (Acu‐TENS) on the respiratory outcomes of COVID‐19 patients with moderate pulmonary involvement.

**Methods:**

In these three‐blind parallel randomized clinical trials, 84 patients with COVID‐19 admitted to a referral hospital were selected by the convenience sampling method. Participants were randomly assigned to Acu‐TENS (*n* = 42) and control (*n* = 42) groups. The Acu‐TENS group received Acu‐TENS over the EX‐B1 (Dingchuan) acupuncture point for 45 min for four consecutive days, while participants in the control group received no intervention. Participants' respiratory outcomes, including oxygen saturation, vital signs, and the severity of dyspnea, were evaluated before and after each intervention on four consecutive days. In addition, the need for mechanical ventilation on Days 4, 8, and 12 and the disease's outcome (death or survival) were recorded in SPSS software version 16, and finally, data were analyzed using an independent samples *t*‐test.

**Results:**

SpO_2_, the number of patients without the need for mechanical ventilation, and patient survival after the intervention were significantly higher in the Acu‐TENS group compared with the control group (<0.001). However, respiratory rate, heart rate, and the severity of dyspnea after the intervention were not significantly different between the two groups (*p* > 0.05).

**Conclusion:**

The use of Acu‐TENS could improve SpO_2_ as a respiratory outcome of patients with COVID‐19 with moderate pulmonary involvement and it can be used as a therapeutic intervention.

## 1 INTRODUCTION

Patients with COVID‐19 experience different symptoms. However, the main symptoms of COVID‐19 are dyspnea and decreased oxygen saturation (SpO_2_). Even patients may have silent hypoxemia despite the exacerbation of the infection.^1^ According to the guideline provided by the Ministry of Health of Iran on the diagnosis and treatment of COVID‐19, patients with symptoms such as dyspnea, chest pain, with or without fever ≥ 38, SpO_2_ between 90% and 93%, and pulmonary involvement less than 50% are in the moderate respiratory phase. In the initial and mild phases of COVID‐19, respiratory involvement is rarely seen. In addition, in the severe phase of the disease, despite the noninvasive oxygen therapy, the arterial oxygen saturation level is less than 88%, symptoms of respiratory failure occur, and shock and multiple organ failure can also occur.^2^ According to an existing retrospective study, the onset of shortness of breath in these patients is usually 5‐6 days after the symptoms.^3^ But the occurrence of dyspnea and hypoxia in critically ill patients can occur after a week from the onset of the disease.^4^ Nonetheless, its progression to acute respiratory distress syndrome is on average, 2.5 days after the onset of shortness of breath, in which case, it is necessary to admit to the intensive care unit (ICU) and use mechanical ventilation.^3^ Therefore, control and management of respiratory signs and symptoms in patients with COVID‐19 are among the most important therapeutic interventions; because dyspnea and low levels of SpO_2_ increase the risk of death.^5^

Various invasive and noninvasive treatments have been used to manage dyspnea in COVID‐19 patients. One of the invasive methods is mechanical ventilation, while this method can have several complications for patients.^6^ Several studies have used noninvasive interventions to reduce dyspnea in these patients, including prone position,^7^ vague nerve stimulation,^8^ electrical stimulation through the skull,^9^ and inhalation combination oxygen/hydrogen.^10^ One of the noninvasive methods is transcutaneous electrical nerve stimulation of acupoints (Acu‐TENS), which may be a potentially effective treatment with the least side effects compared to other methods for relieving the various symptoms and complications of COVID‐19 patients.^11^ However, it must be acknowledged that this intervention is an invasive method and has several complications such as bleeding, pneumothorax, infection, vasovagal reaction, pain, and other complications that can occasionally be life‐threatening.^12^ However, electrical stimulation can be used instead of a needle to stimulate acupuncture points, which is the percutaneous electrical stimulation in acupuncture points or Acu‐TENS. As one of the most modern methods based on acupuncture, this technique has been employed in several studies.^13^ For example, in studies on the respiratory outcomes of patients with lung diseases, including chronic obstructive pulmonary disease (COPD) and asthma, the effectiveness of this method has been reported in improving the respiratory symptoms and respiratory capacity of patients.^14^, ^15^, ^16^, ^17^, ^18^ However, Öncü and Zincir found that applying Acu‐TENS in patients with COPD in the acute exacerbation phase does not affect respiratory parameters.^19^ It is noteworthy that respiratory diseases have different conditions, and there are possible differences in the effectiveness of the Acu‐TENS method on the respiratory conditions of patients. Further, the incidence of respiratory involvement in COVID‐19 patients is high. Accordingly, the present study aimed to investigate the effect of Acu‐TENS on respiratory outcomes in hospitalized COVID‐19 patients with moderate pulmonary involvement.

## 2 MATERIAL AND METHOD

### 2.1 Study design

A parallel, two‐armed, triple‐blind clinical trial design was applied in this study. The independent variable in this research is Acu‐TENS and the dependent variables are SpO_2_, respiratory rate, and heart rate, the severity of dyspnea, as the respiratory outcome of the disease.

### 2.2 Participants

The participants of this study included patients with COVID‐19 admitted to a hospital in an urban area (Shahroud) of Iran from June to September 2021.

The inclusion criteria for participating in the study were the confirmation of the diagnosis of COVID‐19 based on the polymerase chain reaction or computerized tomography scan findings (Ground‐glass opacities [GGO]) by a physician, moderate pulmonary involvement (SpO_2_ between 90% and 93% and pulmonary involvement less than 50%) according to the guideline of the Ministry of Health of Iran,^2^ and the age range of more than 18 and less than 70 years.

On the other hand, hospitalized patients with the other clinical manifestations of COVID‐19 and no pulmonary involvement, use of bronchodilator before the intervention, history of respiratory disease, history of the therapeutic use of electricity, drug and alcohol abuse, and smoking addiction were some of the exclusion criteria. Moreover, patients suffering from comorbidities such as advanced cancer, liver failure, renal failure requiring dialysis, congestive heart failure, having a cardiac pacemaker and cardiac conduction disorders, as well as having a history of allergies, skin allergies, and skin lesions in the electrode area were excluded from the study. The other exclusion criteria included moderate to severe scoliosis, obesity (body mass index above 35), psychiatric disorders, brain vascular disorders, history of mechanical ventilation before the intervention, and inability to sit in a semi‐sitting position for any reason.

### 2.3 Sample size and sampling

**Figure 1:**
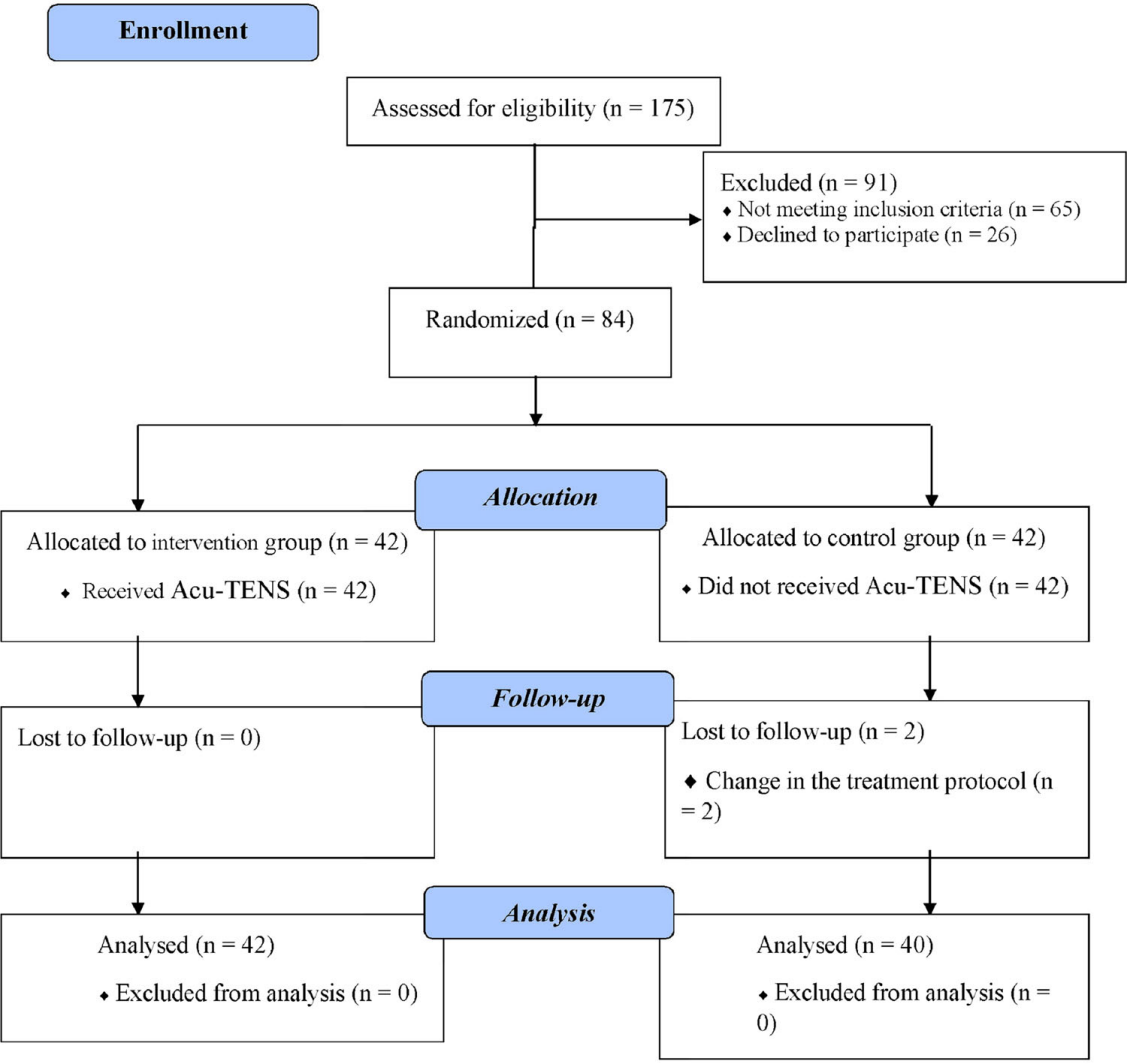
The process of the study according to the CONSORT 2010 flow diagram.

The sample size was estimated to be 42 people in each group considering previous studies, based on the results of mechanical ventilation^20^ and shortness of breath,^21^ and considering the error of the first type of 0.05 and the power of 80%. Convenience sampling was applied to enroll eligible participants in the study. Then, eligible participants were randomly divided into Acu‐TENS and control groups using the quadruple blocking method. Accordingly, after creating a random sequence using SPSS version 16 syntax, a non‐transparent envelope with a wrapper was prepared for allocation concealment.^22^ First, codes “A” and “B” were assigned to the Acu‐TENS and control groups, respectively. Each of the randomly included sequences was recorded on the cards and placed inside the envelopes. The principal investigator registered eligible participants in the order of their arrival, and then each participant was asked to select one of the envelopes. Participants who chose A or B entered the Acu‐TENS or control group, respectively (Figure 1).

### 2.4 Data collection

#### 2.4.1 Demographic data questionnaire

The demographic data questionnaire included data about age, gender, marital status, education level, employment status, place of living, vaccination, and received vaccine dose. The researchers completed the questionnaire at baseline in the COVID‐19 ward.

#### 2.4.2 SpO_2_ and vital sign checklist

The checklist included information on SpO_2_, respiratory rate (RR), and heart rate (HR). The researchers completed the checklist before and after the intervention for four consecutive days in the COVID‐19 ward. The patient's RR and HR were counted in one full minute. In addition, the patient's SpO_2_ was checked using the portable pulse oximetry (made in Iran) linked to a reusable finger sensor probe (Calibration number Q995HE222) without supplemental oxygen on room air. The vital sign was assessed before and after the intervention for four consecutive days in COVID‐19 patients.

#### 2.4.3 The visual analog scale for dyspnea (VASD)

The sensation of dyspnea is subjective, but its severity can be quantified using the visual analog scale. This scale is a simple tool for assessing patients' dyspnea, consisting of a 10‐cm horizontal line rated from 0 to 10; a score of zero indicates no dyspnea, while a score of 10 represents the highest severity of dyspnea. Accordingly, scores 1–3, 4–7, and above 7 demonstrate mild, moderate, and severe dyspnea, respectively.^23^ The severity of dyspnea was evaluated before and after intervention for four consecutive days in COVID‐19 patients.

#### 2.4.4 The need for mechanical ventilation and the disease outcome checklist

Based on the study by Domi et al.,^24^ the time range of the need for mechanical ventilation in patients with COVID‐19 varies between 3 and 12 days after the onset of symptoms. Therefore, the need for mechanical ventilation during Days 4, 8, and 12 was assessed and recorded in the checklist. The outcome of the disease (including death or discharge from the hospital) was also recorded in the checklist.

### 2.5 Intervention

In the Acu‐TENS group, a trained physiotherapist identified the acupuncture point EX‐B1 (Located on both sides of the spinous process of the seventh cervical vertebra); this point has been used in various studies to manage respiratory symptoms.^14^, ^15^, ^16^, ^17^, ^18^ Then, the physiotherapist connected two 5 × 5 cm plastic electrodes at the EX‐B1 acupuncture point, and the electrodes were related to the NOVIN 701 P PLUS‐STIMULATOR (Iranian manufacturer and serial number AX212088). Next, an electric current with a pulse of 200 microseconds and a frequency of 4 Hz Acu‐TENS was applied at the point for 45 min^25^ for four consecutive days.

The device was connected to the front of the chest (unreal treatment point/not related to breathing) for the control group by applying an extremely low‐intensity electric current for 45 min for four consecutive days. It should be noted that the COVID‐19 treatment protocol prescribed by the physician was almost the same in both groups, and all patients received the same treatment.

As mentioned, the intervention was implemented by a trained physiotherapist. One of the researchers collected the data using the mentioned tools without knowing the group of participants. The data collection tool related to each person was coded based on his hospital file number, and then the data was entered into the SPSS software by the researcher's colleague. Due to the accuracy of the collector in collecting and recording data in the tools, no data was missing. In addition to the participants, the data collector who assessed outcomes and the statistical data analyst were unaware of the allocation of the participants.

### 2.6 Data analysis

The results were analyzed using SPSS software version 16. In the statistical tests, a significance level of *p* < 0.05 and a confidence interval (CI) of 95% was used as the *p‐*value. The Shapiro–Wilk test and histogram plot were used to determine the normality of the data. The data were summarized and analyzed using descriptive and inferential statistics (Chi‐square tests, Fisher's exact test, and independent samples *t*‐test, repeated‐measures ANOVA).

### 2.7 Ethical considerations

Ethical approval was obtained from the Ethics Committee of Shahroud University of Medical Sciences (Code: IR.SHMU.REC.1400.008), and the research protocol was registered and approved by the Iranian Registration of Clinical Trials Office (Code: IRCT20210406050871N1). In addition, permission to enter the research environment before the study was obtained from the relevant authorities. Participants were informed of their right to leave the study unpunished, and their information was confidential. A written consent form was obtained from participants who wished to participate in the study.

## 3 RESULTS

In the present study, 175 eligible participants with COVID‐19 were included in the initial study list, of which 91 persons did not meet the inclusion criteria, and 26 people declined to participate in the study. Finally, 84 participants were randomly allocated to two Acu‐TENS (*n* = 42) and control (*n* = 42) groups. During the intervention days, two patients in the control group had attrition due to changes in the treatment protocol, and eventually, the data of 82 participants underwent analysis.

### 3.1 Demographic characteristics and homogeneity comparisons

The mean (SD) of age in the Acu‐TENS and control groups was 56.88 (14.67) and 60.95 (16.45) years old, respectively. The majority of participants in the two groups were married and had primary to secondary education. The number of vaccinated individuals in the Acu‐TENS and control groups was 22 (52.4) and 24 (60), respectively. Other demographic characteristics are reported in Table 1. There was no significant difference between the demographic characteristics of the Acu‐TENS and control groups, and the two groups were homogeneous (*p* > 0.05).

**Table 1:** Demographic characteristics of COVID‐19 patients in the Acu‐TENS and control groups.

Variables	Groups		*p*‐Value
	Acu‐TENS *N* (%)	Control *N* (%)	
Gender			
Male	26 (61.9)	18 (45)	0.12^a^
Female	16 (38.1)	22 (55)	
Marital status			
Single	4 (9.5)	3 (7.5)	0.85^b^
Married	32 (76.2)	32 (80)	
Divorce or death of a spouse	6 (14.3)	5 (12.5)	
Education level			0.70^a^
Primary to secondary education	27 (64.3)	26 (65)	
High school graduate	9 (21.4)	7 (17.5)	
Short‐cycle tertiary education, bachelor's or higher level	6 (14.3)	7 (17.5)	
Employment status			0.75^b^
Unemployed	18 (42.9)	17 (42.5)	
Employed	21 (50)	20 (50)	
Retired	3 (7.1)	3 (7.5)	
Place of living			
City	35 (83.3)	32 (80)	0.92^a^
Village	7 (16.7)	8 (20)	
Vaccination			
Yes	22 (52.4)	24 (60)	0.46^a^
No	20 (47.6)	16 (40)	
Vaccine dose			
One dose	18 (81.8)	18 (75)	0.53^b^
Two doses	4 (18.2)	6 (25)	
Age, mean (SD), year	56.88 (14.67)	60.95 (16.45)	0.25^c^

Abbreviations: Acu‐TENS, transcutaneous electrical nerve stimulation of acupoints; COVID‐19, coronavirus disease 19; SD, standard deviation.

^a^ Chi‐square tests;

^b^ Fisher's exact test;

^c^ Independent samples *t*‐test.

### 3.2 Respiratory outcome

#### 3.2.1 SpO_2_ level

The mean and SD of SpO_2_ levels before and after the intervention and differences in the SpO_2_ level before and after the intervention for 4 days in two groups are provided in Table 2. Based on the comparison of the mean differences in SpO_2_ levels in the first to fourth days before and after the intervention between the Acu‐TENS and control groups, the intervention increased the arterial SpO_2_ level in the Acu‐TENS group (Table 2).

**Table 2:** Respiratory outcome of COVID‐19 patients in the Acu‐TENS and control groups.

Outcome	Intervention days		Groups		*p*‐Value^a^
			Acu‐TENS mean (SD)	Control mean (SD)	
SpO_2_	1 day	Before	92.40 (0.76)	92.21 (0.66)	0.23
		After	95 (1.71)	92.74 (1.51)	<0.001
		The difference before and after	2.59 (1.66)	0.52 (1.55)	<0.001
	2 days	Before	93.17 (1.03)	92.39 (1.424)	0.007
		After	95.98 (1.63)	92.74 (2.36)	<0.001
		The difference before and after	2.80 (1.71)	0.34 (1.77)	<0.001
	3 days	Before	93.93 (1.52)	91.87 (2.01)	<0.001
		After	96.29 (1.77)	92.32 (2.06)	<0.001
		The difference before and after	2.35 (2.47)	0.44 (1.62)	<0.001
	4 days	Before	94.62 (1.37)	92.03 (1.49)	<0.001
		After	97.31 (1.53)	92.00 (1.69)	<0.001
		The difference before and after	2.69 (1.70)	−0.026 (0.88)	<0.001
RR	1 day	Before	16.55 (2.53)	17.58 (1.38)	0.03
		After	16.74 (2.36)	17.71 (1.13)	0.02
		The difference before and after	−0.19 (1.79)	−0.13 (0.70)	0.85
	2 days	Before	16.86 (1.97)	17.16 (1.79)	0.48
		After	16.55 (2.09)	17.37 (1.47)	0.05
		The difference before and after	0.30 (1.45)	−0.21 (1.29)	0.01
	3 days	Before	16.48 (2.42)	17.55 (2.19)	0.04
		After	16.69 (2.50)	17.79 (2.40)	0.05
		The difference before and after	−0.21 (1.70)	−0.23 (1.19)	0.95
	4 days	Before	16.69 (2.20)	18.61 (3.76)	0.006
		After	16.69 (2.45)	18.79 (4.02)	0.006
		The difference before and after	0.00 (1.72)	−0.184 (1.08)	0.57
HR (beat/min)	1 day	Before	78.33 (10.87)	81.71 (9.21)	0.14
		After	79.21 (10.88)	81.42 (10.72)	0.36
		The difference before and after	−0.88 (1.78)	1.60 (12.92)	0.22
	2 days	Before	78.95 (10.81)	82.45 (9.32)	0.13
		After	80.24 (8.88)	81.97 (9.49)	0.40
		The difference before and after	−1.28 (5.52)	0.47 (3.83)	0.11
	3 days	Before	80.14 (8.83)	82.21 (9.23)	0.31
		After	79.26 (8.65)	82.34 (9.69)	0.14
		The difference before and after	0.88 (2.98)	−0.13 (2.23)	0.09
	4 days	Before	79.10 (9.86)	83.84 (10.18)	0.04
		After	79.69 (10.05)	84.29 (10.59)	0.05
		The difference before and after	−0.59 (2.16)	−0.44 (2.34)	0.77

Abbreviations: Acu‐TENS, transcutaneous electrical nerve stimulation of acupoints; COVID‐19, coronavirus disease 19; HR, heart rate; RR, respiratory rate; SD, standard deviation; SpO_2_, oxygen saturation.

^a^ Independent samples *t*‐test.

Repeated measurement analysis was performed on the level of SpO_2_ (before, after, and the difference before and after). In all analyses, the Muchly test is significant, implying that the sphericity assumption is not satisfied; therefore, the Greenhouse‐Geisser statistics were used to examine the effect of time, group, and time‐group interaction.

**Figure 2:**
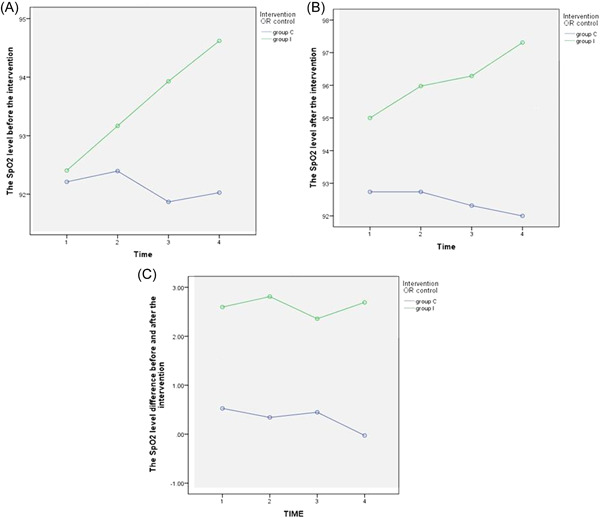
(A) Changes in the SpO_2_ level before the intervention during 4 days in control and Acu‐TENS groups. (B) Changes in the SpO_2_ level after the intervention during 4 days in control and Acu‐TENS groups. (C) Changes in the SpO_2_ level difference before and after the intervention during 4 days in control and Acu‐TENS groups. Acu‐TENS, transcutaneous electrical nerve stimulation of acupoints.

The results revealed that before the SpO_2_ level, the effect of time was significant (*p* < 0.001), representing that there was a difference in the SpO_2_ level before the intervention between 4 days. Additionally, the time‐group interaction was significant (*p* < 0.001) and indicated that before the intervention, the SpO_2_ level had no similar changes in the two groups over time. The effect of the group was significant (*p* < 0.001) and demonstrated that the Acu‐TENS group had a higher SpO_2_ level before the intervention compared to the control group (Figure 2).

It should be noted that the same changes were observed 4 days after the intervention (Figure 2).

Based on the results, for before and after the intervention difference, the effect of time was not significant (*p* = 0.488), indicating that there was no difference in increasing the SpO_2_ between four days. Likewise, the time‐group interaction was insignificant (*p* = 0.158) and indicated that SpO_2_ changes over time for the two groups had similar changes. However, in the analysis, only the effect of the group was significant (*p* < 0.001) and implied that the Acu‐TENS group had more increased SpO_2_ in comparison to the control group (Figure 2).

#### 3.2.2 RR

Comparing the mean (SD) of RR in the first to fourth days before and after the intervention between Acu‐TENS and control groups represented that there was no significant difference between the mean RR before and after the intervention between Acu‐TENS and control groups in the first to fourth days (*p* > 0.05, Table 2).

#### 3.2.3 HR

Based on the mean and SD HR comparison between Acu‐TENS and control groups in the first to fourth days before and after the intervention, the intervention had no significant effect on patients' HR (*p* > 0.05, Table 2).

#### 3.2.4 Severity of dyspnea

Comparing the mean and SD of the score of dyspnea between Acu‐TENS and control groups in the first to fourth days before and after the intervention showed that the intervention did not affect the severity of dyspnea in patients (*p* > 0.05, Table 3).

**Table 3:** The severity of dyspnea in COVID‐19 patients in the Acu‐TENS and control.

Outcome	Intervention days		Groups		*p*‐Value^a^
			Acu‐TENS mean (SD)	Control mean (SD)	
Severity of dyspnea	1 day	Before	5.86 (1.67)	6.63 (1.56)	0.85
		After	6.76 (1.41)	7.29 (1.35)	0.86
		The difference before and after	−0.90 (0.75)	−0.65 (0.53)	0.70
	2 days	Before	6.50 (1.23)	7.26 (1.38)	0.26
		After	6.95 (1.30)	7.61 (1.34)	0.003
		The difference before and after	−0.45 (0.50)	−0.34 (0.48)	0.07
	3 days	Before	6.52 (1.29)	7.26 (1.38)	0.75
		After	7.45 (1.19)	7.95 (1.27)	0.76
		The difference before and after	−0.42 (0.50)	−0.34 (0.48)	0.13
	4 days	Before	7.45 (1.19)	7.95 (1.27)	0.77
		After	8.21 (1.02)	8.63 (1.14)	0.54
		The difference before and after	−0.07 (0.43)	−0.68 (0.47)	0.13

Abbreviations: Acu‐TENS, transcutaneous electrical nerve stimulation of acupoints; COVID‐19, coronavirus disease 19; SD, standard deviation.

^a^ Independent samples *t*‐test.

#### 3.2.5 The need for mechanical ventilation, and the disease outcome

Table 4 presents the frequency of the need for mechanical ventilation and the outcome of the disease in patients with COVID‐19 in both Acu‐TENS and control groups. The results revealed that the number of patients requiring mechanical ventilation on the fourth, eighth, and twelfth days after the intervention in the control group was significantly higher than in the Acu‐TENS group (*p* < 0.05). Further, the number of deaths in the control group significantly increased compared to the Acu‐TENS group (*p* < 0.001).

**Table 4:** The need for mechanical ventilation, and the disease outcome in COVID‐19 patients in the Acu‐TENS and control groups.

Outcomes		Groups		*p*‐Value
Use ventilator		Acu‐TENS *N* (%)	Control *N* (%)	
4 days	Yes	0 (0)	5 (12.5)	0.02^a^
	No	42 (100)	35 (87.5)	
8 days	Yes	0 (0)	11 (27.5)	<0.001^b^
	No	42 (100)	29 (72.5)	
12 days	Yes	1 (2.4)	12 (30)	<0.001^b^
	No	41 (97.6)	28 (70)	
Outcome				
Death		2 (4.8)	18 (45)	<0.001^b^
Live		40 (95.2)	22 (55)	

Abbreviations: Acu‐TENS, transcutaneous electrical nerve stimulation of acupoints; COVID‐19, coronavirus disease 19.

^a^ Fisher's exact test,

^b^ Chi‐square test.

## 4 DISCUSSION

This study was conducted to determine the effect of Acu‐TENS on the respiratory outcomes of patients with COVID‐19 with moderate pulmonary involvement admitted to the hospital. The findings demonstrated that Acu‐TENS during the first to fourth days was effective in improving the SpO_2_ of these patients. However, the intervention in the Acu‐TENS group was not effective on other respiratory outcomes, including RR, HR, and the severity of dyspnea on any of the intervention days. Conversely, the follow‐up of the patient's condition until the last day of hospitalization showed that the intervention had a significant effect on the need for mechanical ventilation during Days 4, 8, and 12 (from the beginning of the intervention) and reduced the need for mechanical ventilation in the Acu‐TENS group compared with the control group. Further, the mortality rate was significantly lower in the Acu‐TENS group compared with the control group.

The results of the present study indicated an increasing trend in SpO_2_ in the Acu‐TENS group after receiving the intervention from the first to the fourth day. Similarly, Ngai et al.^14^ reported that the use of Acu‐TENS five times a week for four consecutive weeks (20 sessions) in patients with COPD resulted in a lower decline in SpO_2_ after a 6‐min walk distance. Contrarily, Liu, et al.,^17^ following Acu‐TENS intervention for 2 days a week for four consecutive weeks on COPD patients, observed no significant effect on SpO_2_. It should be noted that in the above‐mentioned study, several other acupuncture points were used in addition to the EX‐B1 acupuncture point.

In the current study, the RR of patients before the intervention was in the normal range, and the intervention did not affect RR. However, in the control group, fluctuations in the number of breaths were observed more than in the Acu‐TENS group. In contrast, in the study by Ngai et al.,^14^ RR in the intervention group was significantly reduced after 4 weeks (20 sessions of Acu‐TENS intervention). Longer intervention time and longer follow‐up (4 weeks compared to 4 days in our study) could be the reason for this difference in the results. Although one of the critical components of controlling vital signs is checking the RR; changes in the RR are occasionally unrelated to disease conditions such as heart and lung diseases and are sometimes mismeasured.^25^ Studying animal models, Verhoeven et al.^26^ found that SpO_2_ was a reliable indicator of the severity of lung involvement with viral infections, and an increase in SpO_2_ could better demonstrate the process of lung recovery from pathological conditions. Therefore, pulse oximetry can be a good alternative instead of controlling the RR.^25^

In the present study, the HR of patients before the intervention was almost normal, and the intervention did not affect this parameter. However, the results of the study by Jones and Ngai^27^ showed that healthy individuals received one Acu‐TENS intervention before exercise, and their HR faster returned to resting HR after exercise. It is noteworthy that in the mentioned study, healthy individuals without a history of respiratory tract infection during the past 2 weeks were selected, and electrical stimulation was applied through the PC6 acupuncture point (a site often used to manage cardiac symptoms).

It is necessary to mention that the patients did not experience any improvement in dyspnea during the 4 days of the intervention, thus the severity of dyspnea changed from moderate to severe at the beginning of the intervention at the end of 4 days of intervention in both Acu‐TENS and control groups. Likewise, Öncü and Zincir^19^ concluded that Acu‐TENS intervention does not affect dyspnea in patients with COPD in the acute exacerbation phase. However, previous studies on patients with COPD and asthma confirmed a reduction in the severity of dyspnea after Acu‐TENS intervention.^15^, ^16^, ^17^, ^18^ It should be noted that the present study was performed on hospitalized patients who were in the acute phase of the disease, which is similar to the study by Öncü and Zincir.^19^ Dyspnea is a mental feeling affected by conditions such as weakness, anemia, lack of sleep, fatigue, fear, and anxiety,^28^ and physically healthy people may have experienced dyspnea.^29^ Hence, conditions such as COVID‐19, which is associated with high levels of death anxiety^11^ and respiratory muscle weakness,^30^ may cause dyspnea in these patients even without being associated with the worsening of the patient's respiratory conditions. In a study by Leander et al.,^31^ anxiety was reported as the most critical predictor of dyspnea attacks during the rest of the patients with asthma.

Finally, the follow‐up of patients at 4, 8, and 12 days after the intervention showed that the number of patients requiring mechanical ventilation in the Acu‐TENS group was less than that of the control group. In addition, the prognosis of survival was higher in the Acu‐TENS group compared with the control group, and about half of the patients in the control group lost their lives. Patients with COVID‐19 who require intubation and mechanical ventilation due to complications of mechanical ventilation, including atelectasis, respiratory muscle paralysis, and lung dysfunction, have a worse prognosis in comparison to patients without the need for mechanical ventilation.^32^ In this regard, in a study conducted in Iran, the mortality rate of people admitted to the ICU due to COVID‐19 was reported to be 55.6%, which is more than other patients.^28^

One of the leading causes of death in patients with COVID‐19 is lung dysfunction because activated defense mechanisms and the fight against the virus cause lung damage and dysfunction.^29^ In this disease, cytokine storm causes conditions such as acute respiratory distress syndrome and failure in many organs, thus adjusting these conditions is a necessary treatment and rescues these patients.^11^ Traditional Chinese medicine, especially acupuncture, seeks to balance the yin and yang, thus eliminating factors that upset the balance of the body environment due to pathological conditions.^30^ In general, the mechanism of action of Acu‐TENS is still unknown. However, its effectiveness is probably due to stimulating endorphin secretion and inducing a decrease in airway resistance, resulting in easier breathing and inhibiting inflammatory cell release.^17^

### 4.1 Implications for practice

The most critical tasks of healthcare providers include evaluating the patient's respiratory condition, quickly identifying dyspnea, and performing an intervention to relieve an anxious feeling even in mild amounts.^33^ Based on the present study's findings, Acu‐TENS improved SpO_2_ and patients' survival and decreased mechanical ventilation. Accordingly, this method can be used as one of the effective interventions to improve the respiratory outcome of COVID‐19 during the pandemic since it is noninvasive and low‐cost and can be performed by trained healthcare providers.

### 4.2 Strength and limitations

One of the strengths of this research is its design as a randomized clinical trial, which is considered a gold standard for evaluating the effectiveness of the intervention. Another strength of this research was the use of a noninvasive, acceptable available complementary medicine method in controlling the respiratory problems of patients with COVID‐19. However, there were some limitations in this research; the VASD is completed based on the patient's perception of shortness of breath, thus the participant's condition may be effective in completing it; therefore, it was attempted to provide favorable conditions for the intervention and to complete the tool for the patient to minimize bias in this point. In addition, the COVID‐19 infectious disease pandemic has placed many restrictions on patient contact and treatment and care interventions. However, in all stages of the therapist's presence and data collection, efforts were made to pay attention to standard protocols to prevent the spread of the disease. Finally, due to the small sample size, the generalizability of the findings of this study is limited, and the study needs to be replicated by other studies.

### 4.3 Conclusion

The Acu‐TENS could improve SpO_2_ as a respiratory outcome of patients with COVID‐19 with moderate pulmonary involvement. Since the control of oxygen saturation is an essential element in the management of COVID‐19 patients and hypoxemia can lead to many acute adverse effects on their organ systems; the Acu‐TENS can be used as one of the interventions to improve the respiratory outcome of the disease.

## AUTHOR CONTRIBUTIONS

**Amin Shahdad**: Conceptualization; investigation; writing—original draft; writing—review & editing. **Nasrin Fadaee Aghdam**: Conceptualization; investigation; writing—original draft; writing—review & editing. **Shahrbanoo Goli**: Formal analysis; methodology; writing—original draft. **Ehsan Binesh**: Investigation; methodology; writing—original draft. **Javad Nourian**: Investigation; writing—original draft. **Mahboobeh Khajeh**: Conceptualization; methodology; project administration; supervision; writing—original draft; writing—review & editing.

## CONFLICTS OF INTEREST STATEMENT

The authors declare no conflict of interest.

## ETHICS STATEMENTS

The authors acknowledge that the article has not been published elsewhere and is not under review by any other journal. All authors have read the article and agree with its content. The research council affiliated with Shahroud University of Medical Sciences accepted the research protocol, and fully corroborated its ethical consideration, which conforms to the provisions of the Declaration of Helsinki in 1995, revised 2001. As well as the participants provided their written informed consent to participate in this study.

## TRANSPARENCY STATEMENT

The lead author Mahboobeh Khajeh affirms that this manuscript is an honest, accurate, and transparent account of the study being reported; that no important aspects of the study have been omitted; and that any discrepancies from the study as planned (and, if relevant, registered) have been explained.

## Data Availability

All authors have read and approved the final version of the manuscript, and Mahboobeh Khajeh had full access to all of the data in this study and takes complete responsibility for the integrity of the data and the accuracy of the data analysis. Thus, the data that support the findings of this study are available from the corresponding author upon reasonable request. The data that support the findings of this study are available from the corresponding author upon reasonable request.
